# Multiple complications of a well-known disease: a case report of acquired Gerbode defect after bicuspid aortic valve endocarditis

**DOI:** 10.1093/ehjcr/ytad286

**Published:** 2023-06-28

**Authors:** Sara Couto Pereira, Pedro Silverio Antonio, Joana Rigueira, Ana G Almeida

**Affiliations:** Cardiology Division, Heart and Vessels Department, Centro Hospitalar Universitário de Lisboa Norte, E.P.E., Av. Prof. Egas Moniz MB, 1649-028, Lisbon, Portugal; Cardiology Division, Heart and Vessels Department, Centro Hospitalar Universitário de Lisboa Norte, E.P.E., Av. Prof. Egas Moniz MB, 1649-028, Lisbon, Portugal; Cardiology Division, Heart and Vessels Department, Centro Hospitalar Universitário de Lisboa Norte, E.P.E., Av. Prof. Egas Moniz MB, 1649-028, Lisbon, Portugal; Cardiovascular Centre of the University of Lisbon (CCUL), CAML, Lisbon School of Medicine, University of Lisbon, Av. Prof. Egas Moniz MB, 1649-028, Lisbon, Portugal; Cardiology Division, Heart and Vessels Department, Centro Hospitalar Universitário de Lisboa Norte, E.P.E., Av. Prof. Egas Moniz MB, 1649-028, Lisbon, Portugal; Cardiovascular Centre of the University of Lisbon (CCUL), CAML, Lisbon School of Medicine, University of Lisbon, Av. Prof. Egas Moniz MB, 1649-028, Lisbon, Portugal

**Keywords:** Acquired Gerbode defect, Bicuspid aortic valve, Case report, Complete atrioventricular block, Infective endocarditis

## Abstract

**Background:**

Infective endocarditis is a rare but serious disease with high morbidity and mortality due to its potential life-threatening complications. Gerbode defect is an anomalous connection between the left ventricle and the right atrium that can be either congenital or acquired, with previous rare reports following abscess formation in infective endocarditis.

**Case summary:**

A 27-year-old woman presented in hospital with Janeway lesions, stroke, splenic and hepatic abscesses, and transient complete auriculoventricular block. Bicuspid aortic valve infective endocarditis to methicillin-sensitive *Staphylococcus aureus* and acquired Gerbode defect were diagnosed. After intravenous antibiotics and aortic valve replacement, the patient was discharged without sequelae.

**Discussion:**

Bicuspid aortic valve patients have a higher risk of infective endocarditis than the general population. Infective endocarditis may present with multiple complications, including systemic embolization and local perivalvular lesions. Acquired Gerbode defect is a rare complication of infective endocarditis where transoesophageal echocardiography plays an important role for small shunt detection before surgical intervention.

**Learning Points**

- Be aware of the multiple complications of infective endocarditis, currently less frequently encountered in clinical practice due to better prevention and timely diagnosis and treatment.
- Recognize acquired Gerbode defect as a possible complication of infective endocarditis and the importance of transoesophageal echocardiography for its diagnosis.

## Introduction

Infective endocarditis (IE) is a potentially life-threatening endocardial infection, affecting 10 per 100 000 persons annually.^1,2^ Bicuspid aortic valve (BAV) is the most common cardiac congenital abnormality, with a prevalence of 0.5–2%.^3^ According to the public health preventive guidelines, these patients have an intermediate risk of IE, and therefore, antibiotic prophylaxis is not recommended.^4^

Gerbode defect is a rare abnormal communication between the left ventricle (LV) and right atrium (RA) that can cause a LV-to-RA shunt.^5^ This abnormality can be either congenital or acquired after a cardiac insult as myocardial infarction, IE, blunt chest trauma, or previous cardiac surgery.^6,7^ An increasing number of case reports regarding acquired Gerbode due to IE had been published, including in BAV.^8–10^

## Timeline

**Table:** 

**5 days prior the admission**	Fever, chills, asthenia, and headache
**At admission**	Janeway lesions (*Figure 1*) Sudden onset of left hemiplegia and dysarthria Brain CT: deep ischaemic lesion in the right hemisphere Holodiastolic murmur II/VI and no signs of acute heart failure Transoesophageal echocardiography: BAV type 2 without raphe (Schaefer classification), vegetation, and acquired Gerbode defect (*Figure 3*, Video 1–3) Blood cultures: methicillin-sensitive *Staphylococcus aureus* (MSSA). Started antibiotic therapy
**Day 1**	Transient complete atrioventricular (AV) block (*Figure 2B*): temporary transvenous pacemaker implanted
**Day 2**	Abdominal pain and increased transaminases Abdominal CT: liver and spleen abscesses (*Figure 4B*) and bilateral renal ischaemic lesions (*Figure 4C*)
**Day 3**	Control brain CT: Increased ischaemic area and haemorrhagic transformation (*Figure 4A*) Cardiac surgery deferred
**Day 8**	Temporary transvenous pacemaker removed (complete recovery from complete AV block).
**Day 25**	Brain CT: improvement of ischaemic lesions and attenuation of haemorrhagic signs. Abdominal CT: improvement of hepatic and splenic abscesses and renal ischaemic lesions
**Day 31**	Aortic valve replacement with a bioprothesis and acquired Gerbode defect repair
**Day 45**	Discharged

## Case presentation

A 27-year-old woman with known BAV without valvular dysfunction presented in the emergency room with fever, chills, asthenia, and headache that started 5 days prior. She had no current chronic medication, denied intravenous drug abuse, and recent dental interventions.

**Figure 1:**
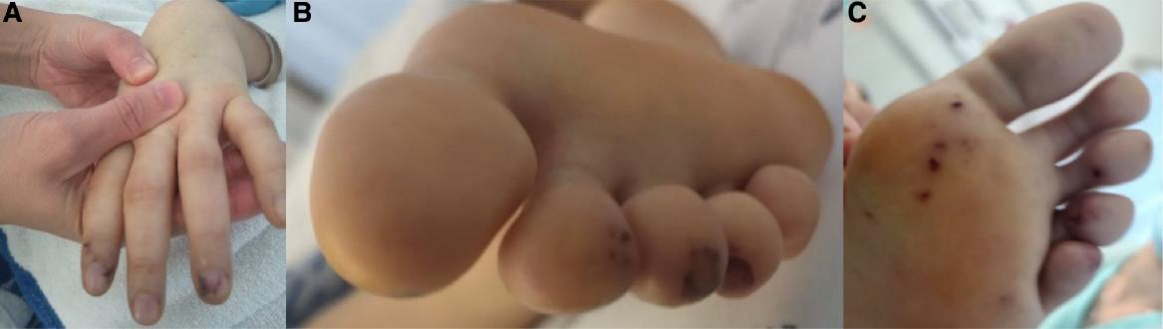
Janeway lesions: (*A*) fingers, (*B*) toes, and (*C*) plantar lesions.

The patient was haemodynamically stable (blood pressure: 112/70 mmHg), tachycardic (110 b.p.m.), and febrile (38.5°C), without respiratory distress. A holodiastolic murmur II/VI and erythematous skin lesions in fingers and toes (*Figure 1*) were found, without signs of acute heart failure (HF).

Laboratory results showed leucocytosis [13.4 × 10^9^/L; normal range (NR): 4.0–11.0 × 10^9^/L], neutrophilia (12.0 × 10^9^/L; NR: 1.9–7.5 × 10^9^/L), raised reactive C-protein 27.6 mg/dL (NR: <0.5 mg/dL), procalcitonin 0.38 ng/mL (NR: < 0.5), thrombocytopenia (85.0 × 10^9^/L, NR: 150.0–450.0/10^9^/L), TGO 101 U/L (NR: <33 U/L), TGP 60 U/L (NR: <32 U/L), and high sensitivity T troponin 139 ng/L (NR: <14 ng/L). Blood samples for cultures were collected before antibiotic therapy initiation, with posterior isolation of methicillin-sensitive *Staphylococcus aureus* (MSSA).

**Figure 2:**
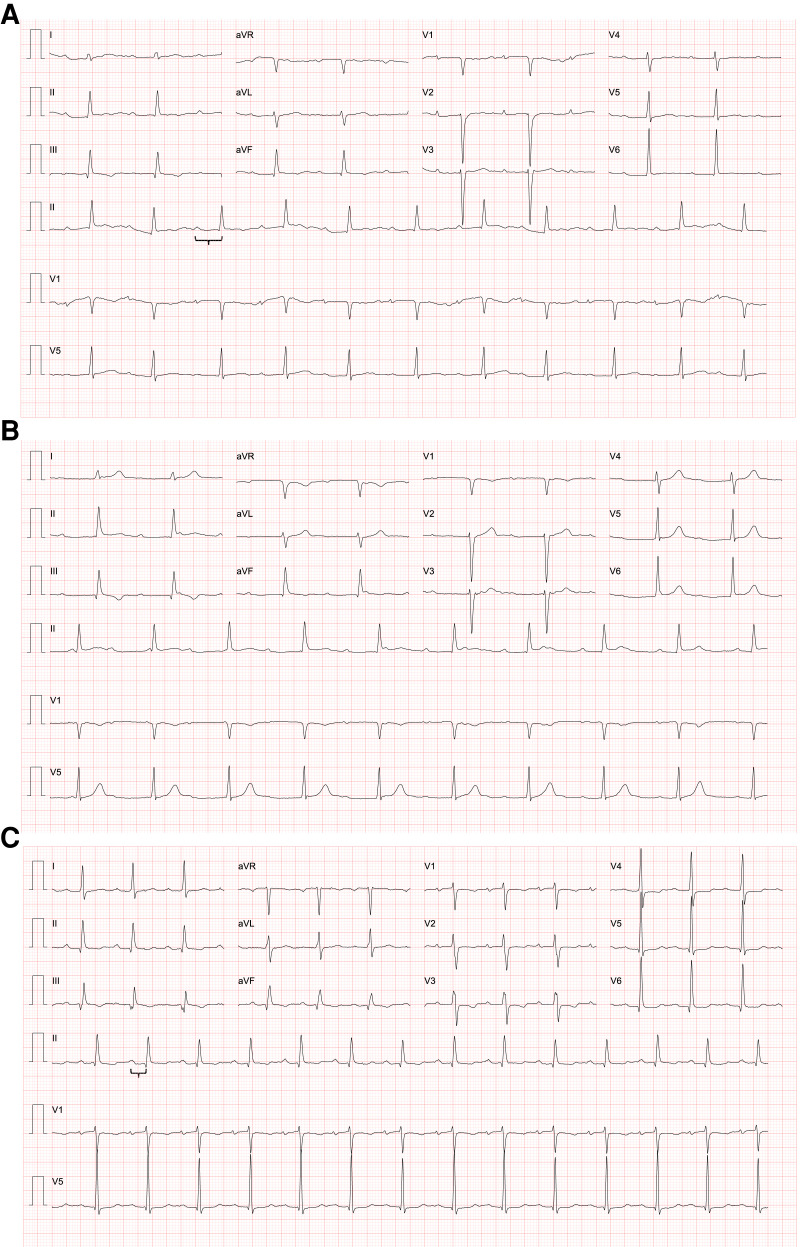
ECG recordings (25 mm/s and 10 mm/mV): (*A*) first-degree AV block at admission, (*B*) third-degree AV block during hospitalization, and (*C*) first-degree AV block at discharge.

Electrocardiogram (ECG) presented *de novo* first-degree atrioventricular (AV) block (PR interval: 335 ms; *Figure 2A*).

During admission, she presented sudden onset of left hemiplegia and dysarthria, with brain computed tomography (CT) showing a deep ischaemic lesion in temporal right hemisphere (right middle cerebral artery).

Transthoracic echocardiogram (TTE) was performed, showing a BAV pattern with mobile filiform mass (22 mm of maximum length) in aortic valve with moderate regurgitation, without obstruction (see Supplementary material online, *Video S1*), non-dilated LV, and preserved LV ejection fraction (LVEF) of 62% (Simpson biplane).

**Figure 3:**
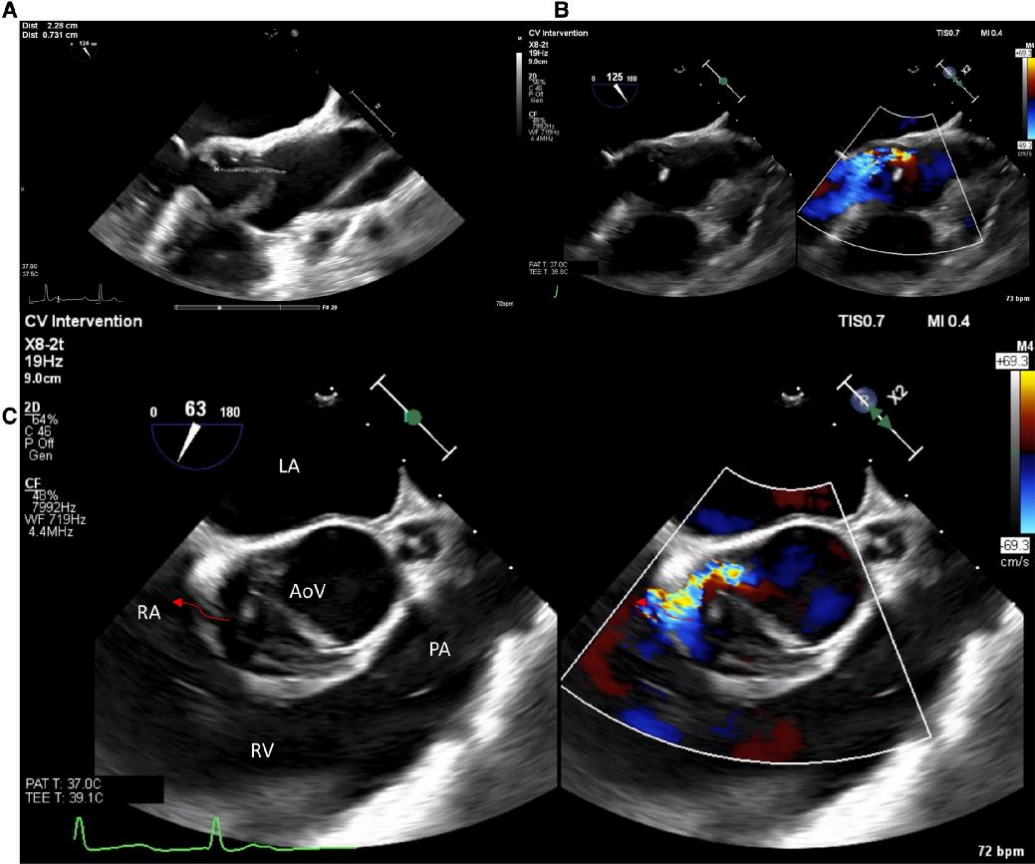
TOE: (*A*) filiform highly mobile mass attached to the mitral–aortic intervalvular fibrosa with dimensions of 22.8 × 7.3 mm, (*B*) moderate aortic regurgitation due to perforation of the posterior/left cusp, and (*C*) LVOT-RA communication (arrow) with high velocity jet at Doppler evaluation (Gerbode defect).

Transoesophageal echocardiogram (TOE) confirmed BAV type 2 without raphe (Schaefer classification), with two mobile vegetations attached to the mitral–aortic intervalvular fibrosa and perforation of the posterior/left cusp (Video 1,2 and *S2*) with moderate regurgitation directed to the anterior leaflet of the mitral valve. A small connection between the membranous portion of interventricular septum at outflow LV tract (LVOT) and RA was found, with LVOT-RA shunt (*Video 3* and see Supplementary material online, *Video S2*; *Figure 3*).

A definite diagnosis of IE was established by the modified Duke criteria. The patient was admitted in the intensive care unit (ICU) for careful monitorization. Empiric intravenous antibiotic therapy was started with ampicillin 12 g/day, ceftriaxone 2 g/day, and flucloxacillin 12 g/day and thereafter adjusted to flucloxacillin 12 g/day (during 31 days before surgery, continued 4 weeks after surgery), rifampicin 900 mg/day (during 24 days before surgery, continued 2 weeks after surgery), and linezolid 12 g/day (during 28 days before surgery, continued 2 weeks after surgery). Routine laboratorial tests and bedside echocardiogram were performed as needed.

**Figure 4:**
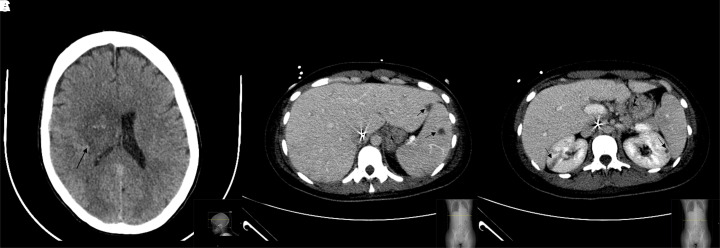
(*A*) Brain CT scan showing deep ischaemic lesion in temporal right hemisphere, with an increased density in the middle (black arrow), compatible with petechial non-confluent haemorrhagic transformation, and mass effect; (*B*) hepatomegaly with small hypodense nodules in the liver and spleen (black arrow), compatible with small abscesses; and (*C*) bilateral renal wedge-shaped hypodense lesions (black arrow), corresponding to small areas of infarction.

At Day 1 after admission, she presented transient complete AV block (*Figure 2B*), with fully recovery after 7 days. An abdominal and pelvic CT scan was performed due to laboratorial abnormalities and abdominal pain, diagnosing systemic embolization to the liver and spleen, with abscess formation and bilateral renal ischaemic lesions (*Figure 4B* and *C*).

Control brain CT showed right petechial non-confluent haemorrhagic transformation (*Figure 4A*), without neurological worsening. Cardiac surgery was deferred during 4 weeks. During hospitalization, the patient recovered from dysarthria and showed a significant improvement of the left hemiparesis.

Until the scheduled surgery, the patient remained haemodynamically stable, without HF signs, preserved LVEF, and without aortic regurgitation worsening. Control blood cultures were negative after 3 weeks of antibiotics.

Thirty-one days after the admission, she underwent aortic valve replacement (AVR) with a bioprosthetic aortic valve (27 mm), as the patient wished. Acquired Gerbode defect was repaired with direct suture using pledgeted prolene on its ventricular face, without post-intervention residual shunt. Aortic valve and pericardium cultures were negative.

After 45 days of hospitalization, the patient was discharged to outpatient follow-up consultation at 3 months and 1 year after. In both follow-up consultations, the patient remained asymptomatic, without clinical events, and had a preserved bioprothesis function.

## Discussion

We described a case of a young woman with BAV without previous documented significant valvular dysfunction that presented with aortic valve IE. This case illustrates the multiple potential life-threatening complications of IE that should be careful managed, including a rare case of acquired Gerbode defect.

The incidence of IE in native BAV patients is higher than that in the general population, usually presenting at younger age, with higher rates of perivalvular complications (abscess formation, valve perforation, and destruction), frequently requiring surgical intervention and exposing patients to more adverse outcomes.^11,12^

*Staphylococcus aureus* is usually responsible for destructive IE, playing an important role in fistula formation.^13^ According to Prifti et al., in 21 cases of acquired Gerbode due to endocarditis, *S. aureus* was the agent in 7, mostly involving the aortic valve and the mitral–aortic intervalvular fibrosa into the upper part of the interventricular septum.^14^ The patient presented perforation of the posterior cusp of the aortic valve, and the two identified vegetations were in continuity with mitral–aortic intervalvular fibrosa, where acquired Gerbode defect was found. Possibly, the fistula resulted from a ruptured abscess.

There are three types of acquired Gerbode defect: (i) supravalvular defect superior to the septal leaflet of the tricuspid valve and involving the membranous portion of the septum (direct), (ii) infravalvular defect below the septal leaflet of the tricuspid valve possibly with leaflet perforation (indirect), and (iii) intermediate—both supravalvular and infravalvular septal communication.^15^ Our patient had a supravalvular defect, without involvement of the tricuspid septal leaflet.

Small acquired Gerbode defects are usually asymptomatic and can be easily missed in TTE. Transoesophageal echocardiogram presents a higher sensitivity and specificity than TTE for diagnosis and detection of IE perivalvular complications, including LV-to-RA small shunts. The communication can be visualized with colour Doppler as a high velocity jet through the shunt.^4^ However, larger acquired Gerbode defects may cause more significant LV-to-RA shunt that may provoke severe acute HF, with sudden dyspnoea due to pulmonary congestion, elevated jugular venous pressure, and hypotension mimicking right ventricular (RV) failure or tamponade. Longstanding shunts may cause RV overload, with progressive RV and RA dilatation and HF symptoms.^15^ In our case report, the acquired Gerbode defect was small and the patient had no symptoms of acute HF, even considering the presence of moderate aortic regurgitation.

Although all the present complications represent an indication for early surgical intervention, the cardiac surgery was deferred due to stroke haemorrhagic transformation, according to the European Society of Cardiology (ESC) guidelines in treatment of IE.^4^ However, evolution was benign at medical therapy despite the worst expected prognosis, allowing the uneventful surgical correction. The patient was discharged without sequelae.

## Conclusion

This case report exposes the IE multiple life-threatening complications that may occur, the complexity of its management, and the importance of TOE in detecting perivalvular complications, as acquired Gerbode defect. Its identification is important for surgical planning since an additional correction besides the AVR may be required.

## Supplementary Material

ytad286_Supplementary_Data

## Data Availability

The data underlying this case report are available in the manuscript and in its online supplementary material.

## Lead author biography

Sara Couto Pereira is a cardiologist at Centro Hospitalar Universitário de Lisboa Norte/Santa Maria University Hospital in Lisbon, Portugal. She graduated in Medicine in 2016 at Instituto de Ciências Biomédicas Abel Salazar—University of Porto, Portugal. She started the cardiology internship in 2018, concluded in 2023. The main areas of interest are cardiovascular imaging and cardiac implantable electronic devices. She is currently attending the Master degree in Cardiovascular Imaging at Lisbon School of Medicine—University of Lisbon.

## References

[1] Nishimura RA, Otto CM, Bonow RO, Carabello BA, Erwin JP 3rd, Fleisher LA, et al 2017 AHA/ACC focused update of the 2014 AHA/ACC guideline for the management of patients with valvular heart disease: a report of the American College of Cardiology/American Heart Association Task Force on Clinical Practice Guidelines. J Am Coll Cardiol 2017;70:252–289. 28315732 10.1016/j.jacc.2017.03.011

[2] Østergaard L, Valeur N, Ihlemann N, Bundgaard H, Gislason G, Torp-Pedersen C, et al Incidence of infective endocarditis among patients considered at high risk. Eur Heart J 2018;39:623–629. 29244073 10.1093/eurheartj/ehx682

[3] Masri A, Svensson LG, Griffin BP, Desai MY. Contemporary natural history of bicuspid aortic valve disease: a systematic review. Heart 2017;103:1323–1330. 28490615 10.1136/heartjnl-2016-309916

[4] Habib G, Lancellotti P, Antunes MJ, Bongiorni MG, Casalta J-P, del Zotti F, et al 2015 ESC guidelines for the management of infective endocarditis: the Task Force for the Management of Infective Endocarditis of the European Society of Cardiology (ESC). Endorsed by: European Association for Cardio-Thoracic Surgery (EACTS), the European. Eur Heart J 2015;36:3075–3128. 26320109 10.1093/eurheartj/ehv319

[5] Gerbode F, Hultgren H, Melrose D, Osborn J. Syndrome of left ventricular-right atrial shunt successful surgical repair of defect in five cases, with observation of bradycardia on closure. Ann Surg 1958;148:433–446. 13571920 10.1097/00000658-195809000-00012 PMC1450812

[6] Yuan S-M. A systematic review of acquired left ventricle to right atrium shunts (Gerbode defects). Hellenic J Cardiol 2015;56:357–372. 26429364

[7] Taskesen T, Prouse AF, Goldberg SL, Gill EA. Gerbode defect: another nail for the 3D transesophagel echo hammer? Int J Cardiovasc Imaging 2015;31:753–764. 25680357 10.1007/s10554-015-0620-3

[8] Hori D, Tanaka M, Yamaguchi A, Adachi H. Surgically treated infective endocarditis involving the aortic bicuspid valve and ventricular septum revealing aortic regurgitation and a Gerbode defect. Gen Thorac Cardiovasc Surg 2010;58:255–259. 20449719 10.1007/s11748-009-0524-z

[9] Kretzer A, Amhaz H, Nicoara A, Kendall M, Glower D, Jones M-M. A case of Gerbode ventricular septal defect endocarditis. CASE 2018;2:207–209. 30370384 10.1016/j.case.2018.03.005 PMC6200681

[10] Sunderland N, El-Medany A, Temporal J, Pannell L, Doolub G, Nelson M, et al The Gerbode defect: a case series. Eur Heart J Case Rep 2021;5:ytaa548. 33598621 10.1093/ehjcr/ytaa548 PMC7873810

[11] Kiyota Y, della Corte A, Montiero Vieira V, Habchi K, Huang C-C, della Ratta EE, et al Risk and outcomes of aortic valve endocarditis among patients with bicuspid and tricuspid aortic valves. Open Heart 2017;4:e000545. 28674620 10.1136/openhrt-2016-000545 PMC5471870

[12] Kahveci G, Bayrak F, Pala S, Mutlu B. Impact of bicuspid aortic valve on complications and death in infective endocarditis of native aortic valves. Tex Heart Inst J 2009;36:111–116. 19436803 PMC2676612

[13] Bashore TM, Cabell C, Fowler JV. Update on infective endocarditis. Curr Probl Cardiol 2006;31:274–352. 16546554 10.1016/j.cpcardiol.2005.12.001

[14] Prifti E, Ademaj F, Baboci A, Demiraj A. Acquired Gerbode defect following endocarditis of the tricuspid valve: a case report and literature review. J Cardiothorac Surg 2015;10:115. 26353810 10.1186/s13019-015-0320-z PMC4565022

[15] Saker E, Bahri GN, Montalbano MJ, Johal J, Graham RA, Tardieu GG, et al Gerbode defect: a comprehensive review of its history, anatomy, embryology, pathophysiology, diagnosis, and treatment. J Saudi Heart Assoc 2017;29:283–292. 28983172 10.1016/j.jsha.2017.01.006 PMC5623025

